# The Advancing of Selenium Nanoparticles Against Infectious Diseases

**DOI:** 10.3389/fphar.2021.682284

**Published:** 2021-07-30

**Authors:** Wensen Lin, Junai Zhang, Jun-Fa Xu, Jiang Pi

**Affiliations:** Department of Clinical Immunology, Institute of Laboratory Medicine, Guangdong Provincial Key Laboratory of Medical Molecular Diagnostics, School of Medical Technology, Guangdong Medical University, Dongguan, China

**Keywords:** selenium nanoparticles, infectious diseases, pathogens, anti-infection therapy, nano-immune synergetic strategy

## Abstract

Infectious diseases, caused by the direct exposure of cellular or acellular pathogens, are found to be closely associated with multiple inflammation and immune responses, keeping one of the top threats to human health. As an indispensable trace element, Selenium (Se) plays important roles in antioxidant defence and redox state regulation along with a variety of specific metabolic pathways. In recent decades, with the development of novel nanotechnology, Selenium nanoparticles (Se NPs) emerged as a promising agent for biomedical uses due to their low toxicity, degradability and high bioavailability. Taking the advantages of the strong ability to trigger apoptosis or autophagy by regulating reactive oxygen species (ROS), Se NPs have been widely used for direct anticancer treatments and pathogen killing/clearance in host cells. With excellent stability and drug encapsulation capacity, Se NPs are now serving as a kind of powerful nano-carriers for anti-cancer, anti-inflammation and anti-infection treatments. Notably, Se NPs are also found to play critical roles in immunity regulations, such as macrophage and T effector cell activation, which thus provides new possibilities to achieve novel nano-immune synergetic strategy for anti-cancer and anti-infection therapies. In this review, we summarized the progress of preparation methods for Se NPs, followed by the advances of their biological functions and mechanisms for biomedical uses, especially in the field of anti-infection treatments. Moreover, we further provide some prospects of Se NPs in anti-infectious diseases, which would be helpful for facilitating their future research progress for anti-infection therapy.

## Introduction

Infectious diseases, induced by deadly pathogens like Covid-2019, *Mycobacterium tuberculosis* and *Staphylococcus aureus* (*S. aureus*), are still major threat to public health with high infectivity and mortality worldwide. Current chemotherapeutic methods have contributed largely to the control of infectious diseases, however, the frequent use of antibiotics with low targeting effects always lead to low treatment efficiency and promoted drug resistance (Ji et al., 2016; Huang et al., 2017). Moreover, the biofilms of multidrug resistant bacteria are always resistant to antibiotics, which results in the need of more powerful therapies (Hoiby et al., 2010; Hoiby et al., 2011). Thus, how to enhance the efficiency of current therapeutics against infectious diseases becomes an emerging urgent issue to global public health.

Selenium is a crucial trace element for maintaining human health through the selenoproteins, antioxidant defense, cell signal transduction, immune regulation and other metabolic processes (Labunskyy et al., 2014). Previous studies have reported that the deficiency of selenium is closely associated with the high morbidity of cancer, infectious diseases, and cardiovascular diseases (Rayman, 2012; Hatfield et al., 2014; Liu et al., 2017). The organic and inorganic selenium compounds that are widely served as food additives. However, with a relative narrow safety at the therapeutic dosage, excessive Se intake can result in unexpected toxic effects (Rayman, 2012).

In the past decade, prompted by the rapid nanotechnology developments, selenium nanoparticles (Se NPs) have attracted extensive attention from researchers in biomedical fields due to their exclusive physical, chemical and biological properties (Skalickova et al., 2017; Menon et al., 2018). Compared with traditional organic and inorganic selenium compounds, Se NPs show numerous advantages including low toxicity, high degradability, excellent anticancer, antimicrobial and antiviral activities (Wadhwani et al., 2016; Hosnedlova et al., 2018). Furthermore, in order to kill cancer cells more efficiently, Se NPs can be furnished as delivery carriers to encapsulate drug or biomacromolecules for chemotherapy (Maiyo and Singh, 2017; Guan et al., 2018). Khurana et al. have reviewed the recent progress and potential therapeutic benefits of Se NPs in various oxidative stress and inflammation mediated disorders like arthritis, cancer, diabetes and nephropathy, as well as the discussions of the significance for the pharmacological activity of Se NPs (Khurana et al., 2019). However, the reviews and discussions for the synthesis of Se NPs, and their anti-infection applications remain to be further emphasized.

More interestingly, Se NPs are also capable of targeting macrophages and regulating macrophage polarization to initiate innate immunity for antimicrobial inhibition by regulating the production of cytokines (Pi et al., 2020). Se NPs could also act as an immunomodulatory agents to inhibit tumor growth by enhancing anti-tumor immune responses, such as regulating tumor-associated macrophages and activating specific T cells (Gautam et al., 2017; Hu et al., 2019). These immunological functions further indicate the potential use of Se NPs as immunomodulatory agents for pathogen defense, thus contribute to the immune therapy of infectious diseases. In this review, we summarize the methods of synthesis and bio-activity of Se NPs, followed by the recent progress of Se NPs for anti-infection treatments, which are expected to facilitate their future research progress for anti-infection therapy.

## Synthesis of Selenium Nanoparticles

Compared with traditional organic or inorganic selenium compounds, the chemical structure of Se NPs is more complicated. Numerous factors should be taken into consideration when the Se NPs are designed and synthesized for biomedical application, including size, shape, composition, surface property and dispersion. Thus, it is of vital importance to develop novel Se NPs with controllable size distribution, functional agents, morphological characteristics and surface properties.

Physical, chemical and biological techniques are three most widely used approaches for the synthesis of Se NPs. With vitamin C, sodium sulfite, sodium thiosulfate and hydrazine as commonly reducing agents, Se NPs are always prepared by chemical reduction method, which is considered as the most convenience method for Se NPs preparation. In addition, Hydrothermal method, template method, laser ablation method, and biosynthesis method are also successfully applied for Se NPs preparation.

### Hydrothermal Method for Selenium Nanoparticles Preparation

The hydrothermal synthesis was developed by Niu et al. With some advantages such as low cost, simple and efficient preparation for the synthesis of crystalline selenium nanostructures (Niu et al., 2012). Typically, an optically polished bulk glass made of GeSe3 is placed in a container filled with deionized water. The sample is hydrolyzed at the reaction temperature, with the release of Se atoms and fragments from GeSe3 in the solution where they form a colloidal suspension of amorphous selenium. Then, Se nanospheres can form a more stable hexagonal crystalline phase and the polycrystalline (t-Se) nanospheres can be observed *via* dissolution recrystallization. Se NPs with different diameters ranging from 10 to 1,000 nm can be obtained using this hydrothermal method.

Using similar hydrothermal method, Shar et al. also prepared Se NPs using sodium selenite as a precursor and L-ascorbic acid as reducing and stabilizing agent, which showed very good hexagonal shape with a clean and smooth surface and revealed very narrow size distribution ranging from 100 to 200 nm (Shar et al., 2019). Shin et al. also demonstrated the reduction of sodium selenite to form elemental selenium nanoparticles using cellulose nanocrystal (CNXL) as a reducing and structure-directing agent under hydrothermal conditions, which prepared Se NPs of 10–20 nm in diameter (Shin et al., 2007). These works suggested that hydrothermal synthesis of Se NPs able to produce functional Se NPs with different shapes and diameters, however, most of the prepared Se NPs using this method was aimed for industry application.

### Selenium Nanoparticles Prepared by Template Method

Se NPs can be formed from selenium element in the guidence of some chemical templates as stabilizers. PEG200 was introduced as a kind of template and surface decorator for Se NPs synthesis (Zheng et al., 2012). Firstly, gray Se was dissolved in PEG200 solution at 210–220°C for 15–20 min, followed the addtion of water at a ratio of 1:1. The solution was centrifuged at 10,000 rpm for 10 min and then washed with Milli-Q water for five times to clear the excess PEG. The as-prepared PEG-Se NPs displayed monodisperse and homogeneous spherical structures with an average diameter of about 5 nm.

Based on chemical reductions, template method is most widely used in the preparation of Se NPs. Using different templates, Se NPs with different size, shapes, and surface properties can be obtained. Hu et al. introduced sulfate polysaccharidesv (SPS) as template to prepare Se NPs using a one-step method (Hu et al., 2020). Polysaccharides were dissolved in DMSO and stirred at room temperature overnight to improve their solubility. Then, sulfur trioxide-trimethylamine (STMA) was added to prepare the SPS at a certain temperature. The resultant solution was cooled to room temperature, neutralized with sodium hydroxide and dialyzed against distilled water for 5 days. Sodium selenite and ascorbic acid aqueous solution with 4:1 molar ratio were added to the prepared SPS solution at room temperature. The reaction product was dialyzed against distilled water and freeze-dried to obtain size controlled stable SPS-Se NPs, with diameter ranging from 54.35 to 123.04 nm.

As the mostly widely used method, the reduction mechanism of sodium selenite by ascorbic acid is listed as below:

$$H_{2}\mathrm{Se}O_{3}+2C_{6}H_{8}\mathrm{—Se}↓\mathrm{+2}C_{6}H_{6}O_{6}\mathrm{+3}H_{2}O$$

During the formation of Selenium element, the presence of some templates, also recognized as stabilizers, would lead these Selenium element to form nano-sized particles in aqueous solutions. A lot of chemicals can be used as templates for Se NPs preparation, such as chitosan (Bai et al., 2017), folate (Pi et al., 2013), hyaluronic acid (Zou et al., 2019),polyethylenimine (PEI) (Li et al., 2016)and ferulic acid (Cui et al., 2018b). In terms of the low cost, convenient procedure and controllable size, these template methods thus offer novel design of Se NPs with high efficacy.

### Laser Ablation Method for Selenium Nanoparticles Preparation

Using laser to irradiate the pure selenium pellet in the bottom of microcentrifuge tube, Guisbiers et al. have successfully prepared Se NPs to inhibit the bacteria growth (Guisbiers et al., 2016). With quick pulse duration and high repetition rate, the laser beam was focused on the surface of the selenium pellet. The irradiation time was fixed at 15 min with a wavelength of 355 nm to produce a more stable colloidal solution. Meanwhile, the conical shape of the cuvette helps to reduce the amount of water required in the vessel, remaining enough height of water above the target to prevent evaporation during irradiation. Compared with other methods, laser ablation reveals many advantages for synthesis of Se NPs, including the reduction of contamination with chemical reagents, low cost for equipment and easy collection of the produced nanoparticles.

Using the similar pulsed laser ablation method, 248 and 532 nm lasers were used to produce Se NPs with different size (Altuwirqi et al., 2020). Menazea et al. synthesized polyvinyl alcohol/chitosan doped selenium nanoparticles *via* one step laser ablation route, which significantly improved the antibacterial activity of their pure blend (Menazea et al., 2020). Additionally, Guisbiers et al. firstly introduced the synthesis of Se NPs by femtosecond pulsed laser ablation at 800 nm in de-ionized water (Guisbiers et al., 2017). The obtained Se NPs have been successfully used to inhibit the formation of Candida albicans biofilms as they can easily adhere on the biofilm, then penetrate into the pathogen, and consequently damage the cell structure by substituting with sulfur. These works further indicated that laser ablation method was very suitable for the preparation of anti-bacterial Se NPs.

### Biosynthesis of Selenium Nanoparticles

Physical and chemical methods have widely developed and used in Se NPs synthesis. However, these methods always require specific environments (such as temperature and laser) and unwanted chemical reagents, which may result in environmental pollution and unwanted toxicity of produced Se NPs (Wadhwani et al., 2016). With growing global environment awareness, the development of environmentally friendly green synthesis has received widespread attention in biomedical fields. Nowadays, a large number of plants are reported for Se NPs synthesis, such as hawthorn fruit (Cui et al., 2018a), lemon leaf (Prasad et al., 2013), *Hibiscus sabdariffa* (roselle plant) leaf (Fan et al., 2020), *Clausena dentata* leaf extract (Sowndarya et al., 2017), *Theobroma cacao* L. bean shell extract (Mellinas et al., 2019), gallic acid (GA) from various fruits and plants (Zhou et al., 2016), and so on.

Furthermore, many bacteria and fungi are also found to synthesize Se NPs based on their ability to reduce selenite to elemental selenium, including Rhodococcus aetherivorans BCP1 (Presentato et al., 2018),*Azoarcus* (Fernandez-Llamosas et al., 2016), *Acinetobacter* (Wadhwani et al., 2018), *Enterococcus faecalis* (Shoeibi and Mashreghi, 2017), *Streptomyces* sp. ES2-5 (Tan et al., 2016), *Pseudomonas aeruginosa* ATCC 27853 (Kora and Rastogi, 2016), Edible *Lentinula edodes* (Vetchinkina et al., 2013). Combining the ability of reduction from plants and microbes with eco-friendly biosynthetic techniques, biosynthesis of Se NPs have presented non-toxicity, lower price, higher stability and biocompatibility than physical and chemical methods. Such biogenic Se NPs are displaying great potential for anti-bacterial and anti-cancer treatments.

## Basic Biological Functions of Selenium Nanoparticles

**FIGURE 1:**
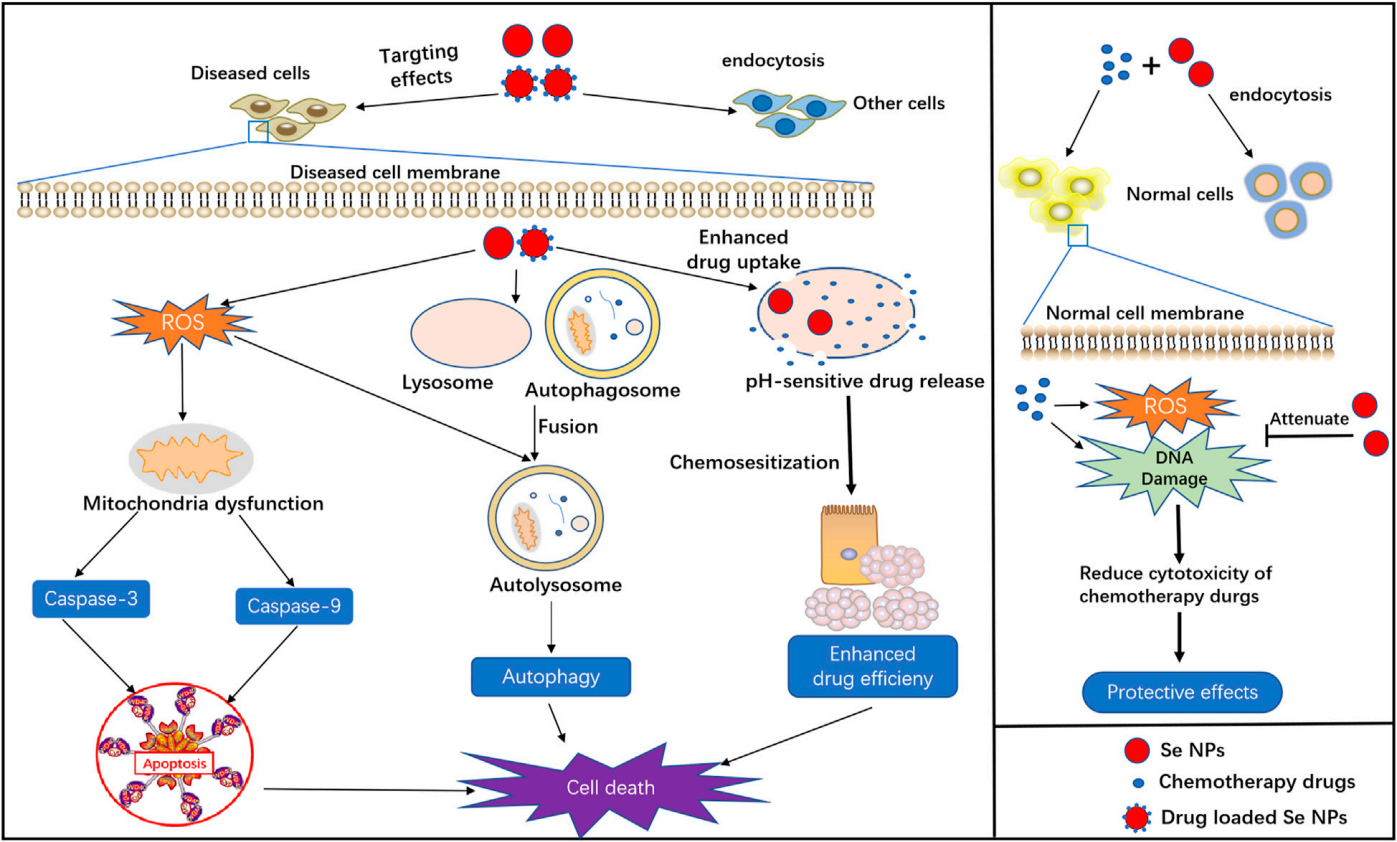
Basic biological functions of Se NPs, including apoptosis and autophagy induction, drug delivery, chemosensitization and protective effects in chemotherapy.

**FIGURE 2:**
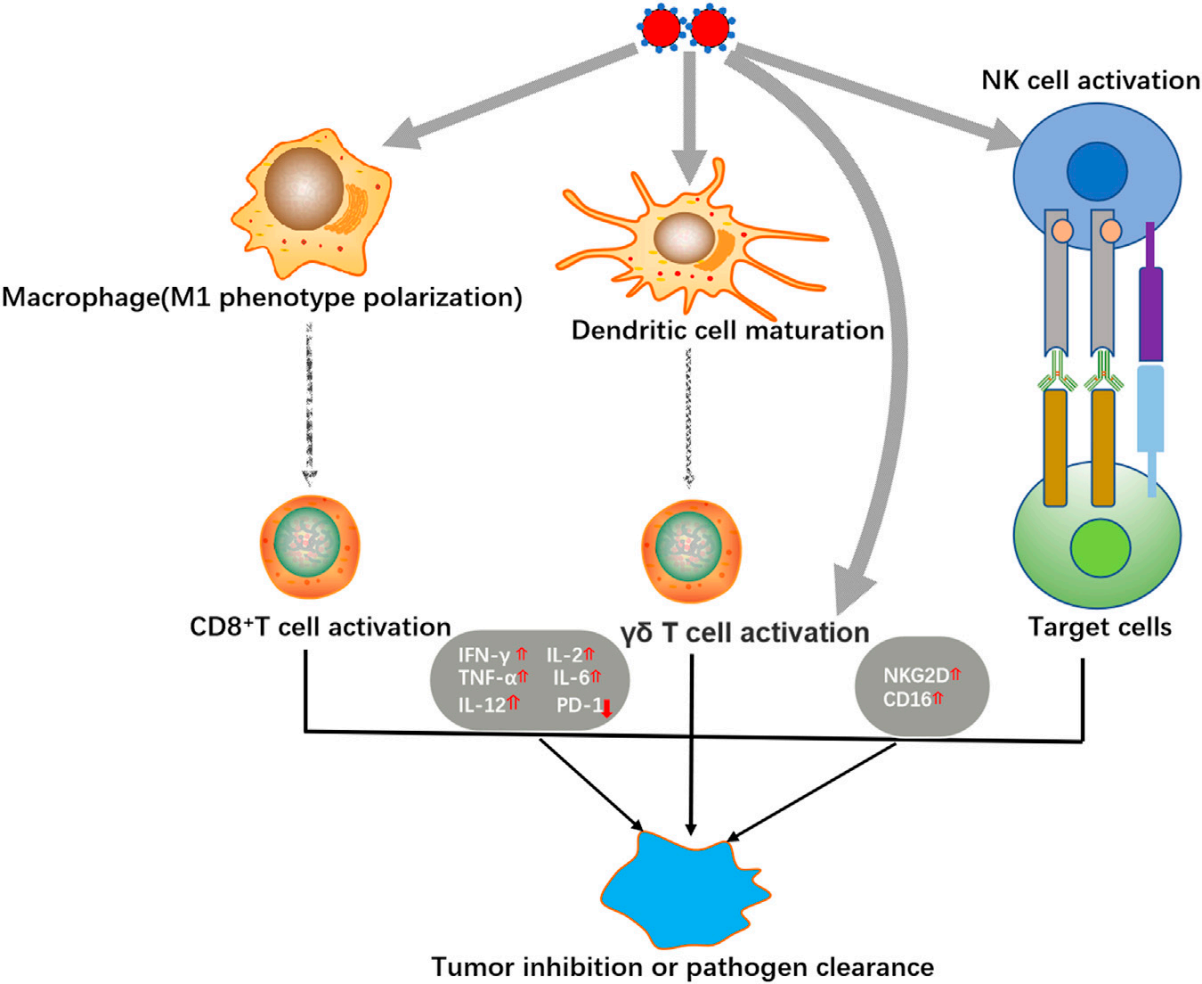
Immunomodulation activities of Se NPs.

As a kind of novel nanomaterials, Se NPs not only display excellent physical and chemical properties, but also exert powerful biological activities for anti-cancer, anti-infection treatments. Herein, we summarized the basic biological functions of Se NPs, such as induce apoptosis and autophagy, act as drug delivery system and protect chemotherapy induced side effects (Figure 1), as well as their immunomodulation effects (Figure 2).

### Selenium Nanoparticles Induced Cell Apoptosis

Apoptosis, an active mode of cell death, is a genetically regulated suicide mechanism that plays an important role in the development and defense of muticelluar organisms (Zhang et al., 2013). Many studies have reported that ROS formation is involved in the process of apoptosis and closely related to the electron respiratory chain of mitochondria. Generally, Se NPs are found to produce excess ROS for apoptosis induction through the blockage of electron respiratory chain. Therefore, the up-regulated intracellular ROS generation induced by Se NPs are capable of inducing cell apoptosis and cell cycle arrest, which is helpful for facilitating cancer cell death. Furthermore, apoptosis can also manifest killing effects towards cancer cells through extrinsic or intrinsic pathways (Menon et al., 2018). The extrinsic pathway can be initiated by attachment of a pro-apoptotic ligand to death receptor, which triggers caspase 8-mediated apoptosis. Whereas, the intrinsic pathway is activated within the cell through caspase 9 due to DNA damage or cell oxidative stress. Thus, excessive ROS production that damages the DNA and induces apoptosis is always responsible for the cytotoxic effects of Se NPs(Wang Y. et al., 2015).

The most widely application of Se NPs induced apoptosis is to kill cancer cells with the participation of some important signaling events, such as ROS generation, anti-apoptotic gene down-regulation, pro-apoptotic gene up-regulation and caspases activation (Nie et al., 2016; Bidkar et al., 2017; Cui et al., 2018b). Internalized Se NPs can quickly and apparently initiate extrinsic signaling pathways such as DISC/caspase-8/caspase-3 signaling, to promote the most common form of physiological cell death-apoptosis. Meanwhile, through the cell cycle analysis, Se NPs are also found to induce apoptosis with the involvement of G2/M phase arrest in a dose-dependent manner. Moreover, overproduction of ROS could lead to mitochondria dysfunction, such as the disruption of mitochondria membrane potential (MMP), which may contribute to the activation of mitochondrial apoptosis pathway. The overproduced ROS could always increase cytochrome c to induce the activation of caspase-9, which acts as the vital pro-apoptotic protein and promotes the downstream caspase-3 activation, leading to the intrinsic apoptosis. Such strong ability to kill cancer cells with low cytotoxicity against normal cells by Se NPs, would contribute to the inhibition of *in vivo* tumor growth (Xia et al., 2018; Song et al., 2020).

Li et al. investigated the anti-cancer effects and mechanisms of Galangin functionalized Se NPs(Se@Ga) on human hepatoma HepG2 cells (Li et al., 2018b). With relatively high cytotoxicity and anti-proliferative effects, Se@Ga could effectively inhibit HepG2 cell proliferation, which was associated with apoptosis induced by ROS generation. These results provide valuable strategies on anticancer treatment for exploring the mechanism of Se NPs induced apoptosis in HepG2 cells. And using transferrin (Tf)-conjugated Se NPs loading with doxorubicin, Huang et al. demonstrated the strong ability of Tf-Se NPs to activate caspase-3, caspase-8, caspase-9 associated apoptosis (Huang et al., 2013; Wang Y. et al., 2015). Importantly, some anti-apoptotic genes of Bcl-xl and ERK were suppressed, while pro-apoptotic genes of p38, p53 and Bad were up-regulated. Wang et al. found that selenium-substituted hydroxyapatite (HA) nanoparticles could inhibit Cdk1 protein expression, arrest the cell cycle at the S-G2/M phase, and accelerate the DNA damage of cancer cells, leading to tumor necrosis (Yanhua et al., 2016). Additionally, when entering into HCC cells, these prepared Se NPs could be dissolved by lysozyme to release lots of calcium ions that could destroy the cell membrane and facilitate the ROS generation induced cell death.

And except for the ability to kill cancer cells, apoptosis is also a common pathway for the destruction of intracellular bacteria, which is very important for the host immunity defense against bacterial infection (Behar and Briken, 2019). During microbial infection, apoptotic cell death is generally beneficial for the host and detrimental for the pathogen, as it can avoid the pathogens to exit the host cells for further disperse. However, some pathogens have tremendously stable cell walls that are unlikely to be damaged by apoptosis, and some pathogens may also have the ability to inhibit host cell apoptosis as a critical way for their immune escape (Behar and Briken, 2019). Therefore, it would be a great idea to clear the intracellular bacterial pathogens by inducing apoptosis in infected cells, which provides the possibility to enhance host cell immunity by regulating apoptosis (Bewley et al., 2014; Cui et al., 2016; Lee K.-I. et al., 2020). Our recent work also demonstrated the ability of Se NPs to induce Mtb-infected macrophage apoptosis, which was helpful for the antimicrobial immunity and intracellular Mtb clearance and killings (Pi et al., 2020).

### Selenium Nanoparticles Promoted Cell Autophagy

As a catabolic process, autophagy is necessary and beneficial for cell homeostasis as it could prevent the toxic protein aggregation, remove damaged organelles and provide cell and organism with bioenergetic substrates for survival (Doherty and Baehrecke, 2018). However, excessive autophagy that is correlated with cell apoptosis could consume the cellular organelles, causing irreversible disorder of functions in cells and even leading to cell death (Chen et al., 2018).

Autophagy can suppress or promote tumors depending on the developmental stage and tumor type. Modulating autophagy for cancer treatment is an attractive therapeutic strategy currently under intense investigation (Antunes et al., 2018). Apart from the promotion of cell survival, autophagy can also initiate the apoptotic signaling pathways to induce cancer cell death, which provide a novel idea in nanoparticle-induced cytotoxicity. Se NPs have been found to regulate autophagy in different type of cancer cells, which are always associated with cancer cell apoptosis. The intracellular autophagy initiator Beclin-1 was found to be significantly up-regulated to increase LC3-Ⅱ expression and decrease p62 expression in the first 24 h of Se NPs treatment, indicating that Se NPs could facilitate the formation of autophagosome and promote the process of autophagy by regulating autophagy proteins. Besides, autophagy may synergistically promote apoptosis to induce cancer cell death in a time and dose manner upon Se NPs treatment. Excessive activation of autophagy could lead to mitochondrial dysfunction, which would eventually induce apoptosis of tumor cells (Huang et al., 2018; Huang et al., 2019). However, some studies have also demonstrated that Se NPs play a role in inhibiting autophagy to reduce the resistance of tumor cells. The levels of p62 and Beclin-1 was significantly increased after treatment with Se NPs for 12 h, suggesting that the early phase of autophagy was activated but the late phase of autophagy was blocked. These results indicated that autophagy related to the self-production mechanisms of tumor cells was inhibited by Se NPs. Moreover, though the evaluation of lysosomal acidity, Se NPs were found to reduce the fusion between autophagosomes and lysosome or suppressed the degradation of lysosomes, which ultimately inhibited the late stage of autophagy (Cui et al., 2019). Such autophagy promoting or blocking mechanisms in different cell models induced by Se NPs further confirmed the anticancer strategy by regulating cancer cell autophagy.

It’s also important to note that autophagy can regulate immunological functions that influencing pathogen infection and pathogen survive in host cells. The host cells tend to kill the intracellular pathogens by autophagy pathways, but some bacteria have developed diverse strategies to avoid autophagy by interfering with autophagy signaling or the autophagy machinery (Huang and Brumell, 2014). And in some cases, pathogens can even exploit autophagy for their growth (Huang and Brumell, 2014). Thus, how to regulate host cell autophagy for intracellular pathogen clearance remains a big challenge. Some interesting ideas to kill intracellular pathogens by inducing autophagy has presented the attractive prospect of autophagy-associated anti-bacterial strategy (Tindwa et al., 2015; Biering et al., 2017; Lee H. J. et al., 2020). We have also demonstrated the possibility of using Se NPs to promote autophagy in Mtb-infected macrophages, which lead to the enhanced intracellular Mtb inhibition, thus providing novel method for intracellular Mtb clearance (Pi et al., 2020).

### Drug Delivery by Selenium Nanoparticles

Taking the advantages of targeted drug delivery and controlled drug release, functional nanosystems provide novel therapeutic strategies for disease treatment. With low toxicity, high bioavailability and biocompatibility, Se NPs can be conjugated with different kind of agents for targeted drug delivery. Drugs can be loaded into Se NPs at high concentrations than their intrinsic solubility, which significantly enhanced their anticancer effects (Liu et al., 2012; Guan et al., 2018). Compared with individual agents, Se NPs showed higher selectivity to cancer cells with high bioactivity with increased drug solubility and targeting effects, and finally resulted in the increased drug efficiency and reduced side effects (Chen et al., 2008).

Xia et al. used galactose (GA) modified Se NPs as doxorubicin (DOX) delivery system with active tumor-targeting property (Xia Y. et al., 2019). And in order to reduce the side effects of chemotherapeutic drugs, Liu et al. prepared 5-fluorouracil surface-functionalized selenium nanoparticles (5FU-Se NPs), which significantly enhanced anticancer efficacy via the induction of caspase-dependent apoptosis (Liu et al., 2012). Zhang et al. have developed an injectable Se NPs nanosystem based on the thermosensitive hydrogel PLGA-PEG-PLGA to load sorafenib (SOR) as effective drug release library for both *in vitro* and *in vivo* tumor inhibition (Zheng et al., 2019). Our previous works also demonstrated the potential of GE11 peptide conjugated Se NPs as a delivery system to enhance the cancer targeting effects and solubility of oridonin, which dramatically enhanced the anticancer effects of Oridonin both *in vivo* and *in vitro* (Pi et al., 2017).

Additionally, the drug delivery capacity of Se NPs can also be applied to enhance the killing efficiency of drugs against bacteria. Liu et al. introduced ciprofloxacin loaded engineered selenium lipid nanocarriers as effective drug delivery system for preventing lung infections of interstitial lung disease (Liu et al., 2019). Our recent work also introduced the use of Se NPs as macrophage-targeted delivery system to enhance the intracellular Mtb killing efficiency of Isoniazid (Pi et al., 2020), indicating that Se NPs could also serve as drug delivery system for intracellular pathogen clearance.

### Immunomodulation of Selenium Nanoparticles

Se NPs have been proved to exhibit strong immunomodulatory activity by regulating different immune cells or modifying some important immune-associated signaling events (Figure 2). With the rapid development of chimeric antigen receptor T-cell (CAR-T) therapy, immune therapy has emerged as a promising new treatment for malignant tumors (Neelapu et al., 2018). Taking the advantage to strengthen the anti-tumor cytotoxicity of immune cells, Se NPs have been proved to benefit immune therapy of tumor.

Wang et al. fabricated a novel immunogenic core-shell Au@Se NPs to activate anti-tumor immunity by synergetic manipulation of Se NPs-mediated chemotherapy and Au NSs-induced photothermal therapy (Wang J. et al., 2020). The *in vivo* results indicated that Au@ Se NPs not only generated the anti-tumor immune responses with excellent cancer killing effect under the presence of tumor-associated antigens, but also effectively transformed the tumor associated macrophages (TAMs) from M2 to M1 phenotype. These effects could further promote T cell activation for tumor rejection, which also contributed to phagocytosis of the distant tumor. Hu et al. introduced the effects of Se NPs to up-regulate the expression of cytotoxicity related molecules including NKG2D, CD16, and IFN-γ in γδ T cells, meanwhile, downregulate PD-1 expression in γδ T cells, which significantly enhance the cancer killing effects and *in vivo* tumor growth inhibition (Hu et al., 2019). These application of Se NPs for innate and acquired immunity modulation strongly suggest the potential use of Se NPs as immunoregulator against cancer.

Infectious diseases are always associated with multiple immunological responses, thus providing the possibility to treat infectious diseases by modulating immunity. Dietary chitosan-selenium nanoparticle (CTS-Se NP) have been proved to enhance immunity and disease resistance in zebrafish against bacterium *Aeromonas hydrophila* infection (Xia I. F. et al., 2019). With the stimulation of lipopolysaccharide (LPS) and concanavalin A (ConA), zebrafish splenocytes exhibited higher proliferation after treatment of CTS-Se NP. And the immune response of splenocytes against ConA was found to be associated with the up-regulation in IL-2 and IL-12 production. Our work also demonstrated the ability of Se NPs to inhibit Mtb-lysosome escape, and promote the host antibacterial immunity to induce host cell apoptosis, autophagy, and M1 anti-bacterial polarization, which significantly enhanced the intracellular Mtb killing efficiency (Pi et al., 2020). These works collectively suggest that Se NPs could be served as novel immunomodulator against different bacteria infection, which therefore provides new possibilities for infectious diseases treatment.

### Antimicrobial and Antiviral Activity of Selenium Nanoparticles

Se NPs have aroused widespread interest due to their powerful antimicrobial activity. With the enhanced release of selenium ions to destroy the bacteria structure, Se NPs can be used to prevent multidrug-resistant bacterial infections, which therefore shows promising potential as antibiotic alternative (Lin et al., 2019). Moreover, chitosan coupled Se NPs have possessed efficient dose-dependent inhibition against *C. albicans* biofilm, further confirming the fungicidal effects of Se NPs (Lara et al., 2018). Besides, biosynthesized Se NPs could effectively suppress the growth of type-1 dengue virus, indicating the antiviral property of Se NPs (Ramya et al., 2015). These works strongly suggest that Se NPs can be served as a reliable agent to directly anti-bacterial and anti-viral treatment, which we will further discuss in the following section.

### Selenium Nanoparticles Induced Chemosensitization

Drug resistance is a major challenge for cancer and infectious disease therapies, which results in treatment failure. As a kind of novel drug delivery system, Se NPs not only enhance the targeting effects of drugs, but also present the strength to increase the drug sensitivity for anticancer and anti-infection treatment (Liu et al., 2012; Pi et al., 2017; Xia Y. et al., 2019; Zheng et al., 2019; Pi et al., 2020). Ahmed et al. showed that Se NPs could potentiate the cancer inhibition effects of 5-fluorouracil (FU)-encapsulated PLGA nanoparticles for enhanced chemo-sensitivity (Abd-Rabou et al., 2019). These functions were found to be associated with the regulation of antioxidant activity by selenium *via* glutathione peroxidases and thioredoxinreductases, which was considered to be one of the main mechanisms of Se NPs for cancer therapy. An *in vivo* study also indicated that administration of Se NPs along with Cyclophosphamide caused more significant reduction in tumor volume, packed cell volume, viable tumor cell count, and increased the survivability of the tumor-bearing hosts (Bhattacharjee et al., 2017). Such interesting ability of Se NPs thus provide novel possibility to use Se NPs for enhanced anti-infectious diseases treatment, although lots of works need to be done.

### Protective Effects of Selenium Nanoparticles

With low toxicity and high bioavailability, Se NPs have the ability to protect normal cells from the cytotoxic effects of common chemotherapeutic agents. For instance, Rezvanfar et al. have found that Se NPs may be helpful to prevent cisplatin -induced gonadotoxicity through antioxidant capacity (Rezvanfar et al., 2013). With cisplatin and Se NPs treatment, serum testosterone, sperm quality, and spermatogenesis in rats were significantly improved. And cisplatin-induced free radical toxic stress and spermatic DNA damage were also reduced in male rats by Se NPs treatment, which significantly alleviated the toxicity of cisplatin. Li et al. also introduced the use of Se NPs to achieve enhanced antioxidant activity and antagonis against cisplatin-induced nephrotoxicity, which indicated the attractive potential of Se NPs in prevention of cisplatin-induced renal injury (Li et al., 2011). These works suggest that Se NPs could also act as a kind of protective agents in chemotherapy to reduce the side effects, which might provide new solutions for the current strong side effects of antibiotics against infectious diseases.

## Anti-Infection Application of Selenium Nanoparticles

**FIGURE 3:**
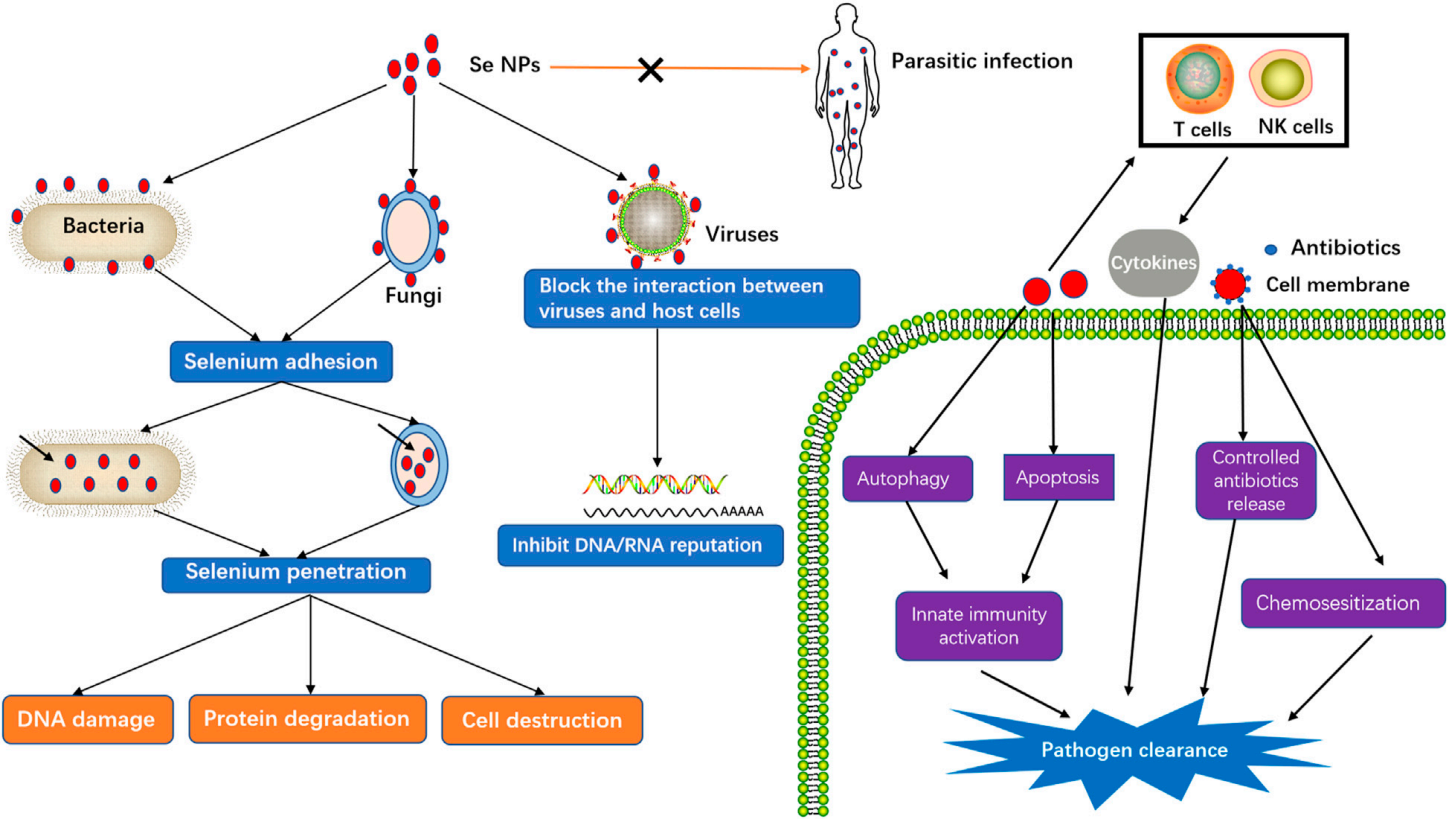
Killing effects of Se NPs against infection.

Taking the advantages of smaller size and higher surface area that can facilitate the reactions with biological molecules, Se NPs have also gained lots of attention for anti-infection treatment. The common mechanism of biofilm disruption is beneficial to inhibit the pathogen growth (Vallet-Regi et al., 2019). Besides, microbial resistance is generally correlated with the cell wall and cell membrane that form a rigid defensive barrier towards environmental aggression. The increased Se ions can not only disrupt the cell walls, but also destroy the integrity of cell membranes, which would result in intracellular homeostasis damage and microbial dysfunction, thereby leading to the death of microbial cells (Baptista et al., 2018). To further understand the emerging roles of Se NPs against infectious disease, we therefore summarized the anti-infection of Se NPs against pathogenic bacteria, fungi, virus and parasite (Figure 3).

### Anti-Bacterial Activity of Selenium Nanoparticles

Bacteria are small organisms that can invade the body, although most of them are harmless, and some actually help to digest food, destroy disease-causing microbes, fight against cancer cells, and provid essential nutrients. However, a small fraction of harmful bacteria are capable of crowding out healthy bacteria, growing in sterile tissues and emiting toxins, which causes complicated bacterial infection diseases. Although most of the bacterial pathogens can be successfully controlled by current antibiotics, some extremely cunning bacteria or drug-resistant bacteria are still serious threatening to human health. As a kind of potent antibacterial agents, Se NPs can inhibit a panel of nosocomial infection caused by pathogenic bacteria (Hariharan et al., 2012), which introduces Se NPs a potential candidate as antibacterial agents or chemosensitizer for enhanced bacteria killing.

### Broad-Spectrum Anti-Bacterial Effects of Selenium Nanoparticles

Selenium status may affect the function of cells in both adaptive and innate immunity, therefore shows the ability of selenium to control bacterial infection. Additionally, selenium compound has also been found to show strong bacteria inhibition effects on different bacterial pathogens (Narayanan et al., 2018) (Pellissery et al., 2019). As a kind of novel selenium form with stronger biological activities than selenium compounds, Se NPs are also widely reported to show wide-spectrum antibacterial activity (Wang Q. et al., 2015; Guisbiers et al., 2016; Liu et al., 2016; Tran et al., 2016; Medina Cruz et al., 2018; Yazhiniprabha and Vaseeharan, 2019). Moreover, owing to the ability to adsorb proteins, restrain the biofilms, disrupt the membrane, regulate bacterial genes and reduce the free intracellular thiol, Se NPs have exhibited efficient antimicrobial properties *in vivo* and vitro studies.

*Staphylococcus aureus* (*S. aureus*), one of the typical Gram-positive bacteria, is the major antibiotic-resistant pathogen inducing skin infections, pneumonia, enteritis and other deadly diseases (Oliveira et al., 2018). It has been proved that *S. aureus* can be significantly killed by Se NPs in few hours, which introduced the possibility of Se NPs for direct Gram-positive bacteria killing (Tran and Webster, 2011). And additionally, the adherence on different surfaces and the biofilm formation of *S. aureus* could also be significantly inhibited by Se NPs (Cihalova et al., 2015; Sonkusre and Singh Cameotra, 2015). These results suggested that Se NPs might also be used for medical device coating to serve as an alternative approach for prevention of biofilm related infections.

Furthermore, Se NPs also showed strong antibacterial activity against Gram-negative such as *Escherichia coli* (*E. coli*). Biogenic Se NPs without any cytotoxicity exerted protective effects on intestinal barrier dysfunction caused by Enterotoxigenic E.*coli* K88, which played an essential role in promoting the growth of intestinal epithelial cells and maintaining intestinal microflora balance (Xu et al., 2018). These inhibition effects of Se NPs against E.*coli* might be closely associated with the ability of Se to reduce exopolysaccharide (EPS) synthesis, inhibit biofilm formation, and inactivate the mature *E. coli* biofilms (Nair et al., 2018).

Besides, some other Gram-positive bacteria, including *Staphylococcus epidermidis* (Tran et al., 2019), *Bacillus substilis* (Chandramohan et al., 2019), *Enterococcus faecalis*, *Streptococcus mutans* (Yazhiniprabha and Vaseeharan, 2019), and some other Gram-negative bacteria including *Pseudomonas aeruginosa* (Srivastava and Mukhopadhyay, 2015; Liu et al., 2019) can also be suppressed by Se NPs, which strongly suggested the wide-spectrum antibacterial activity of Se NPs.

### Drug-Resistant Bacteria Inhibition by Selenium Nanoparticles

The threat of antimicrobial resistance is a worsening problem in recent decades not only in public health, but also in economic and social impacts, which requires the development of new drugs for more effective treatments. Nanomedicines have attracted increasing attentions for fighting against bacterial resistance to offer a chance of biofilm internalization, prolong antibiotic release, increase targeted delivery effects and improve systemic circulation of antibiotics (Eleraky et al., 2020). The strong ability of Se NPs to inhibit bacterial growth also provide new strategies against drug-resistant bacteria infections.

When combined with some antibacterial components, Se NPs would show more potent antibacterial activities. Lysozyme is a biomolecule that has been widely distributed in humans, vertebrates, plants, bacteria and phages, which plays an important defensive role in the innate immune system and direct bacteria killings. Considering the natural antimicrobial effects of lysozyme, as well as the promising antimicrobial potential of Se NPs, Vahdati et al. investigated the interactions between Se NPs and lysozymes, and also determined their combined anti-bacterial effects (Vahdati and Tohidi Moghadam, 2020). Se NPs play an important role in inhibition of bacterial growth at very low concentrations of lysozyme, whereas very high amount of the lysozyme is required to inhibit bacterial growth individually. These results indicate the potentials to design Se NPs-based nanohybrid systems with synergistic antibacterial properties to overcome the emerging antibiotic resistance as well as to define fruitful applications in biomedicine and food safety.

Huang et al. reported that Se NPs exhibited strong antibacterial activity against eight bacterial species, including Gram-positive, Gram-negative, and drug-resistant strains (Huang et al., 2020). Furthermore, unlike the conventional antibiotic kanamycin, Se NPs did not readily induce resistance in *E. coli* or *S. aureus*, indicating the potential use of Se NPs to delay drug resistance. Lin et al. established a kind of novel Se NPs system for methicillin-resistant *Staphylococcus aureus* (MRSA) treatment combining the advantages of natural red blood cell membrane (RBCM) and bacteria-responsive gelatin nanoparticles (Lin et al., 2019). The obtained Se NPs were helpful for the escape of nanoparticles from the immune clearance and neutralization bacterial exotoxins. gelatin nanoparticles could be degraded by gelatinase in pathogen-infected areas *in situ* for controlled Se NPs release, which could induce robust ROS generation to destroy bacterial cell membrane effectively.

These works suggest that Se NPs might be a promising antibiotic alternative to combat different kinds of bacteria, including the threatening multidrug-resistant bacteria. The strong effects of Se NPs to destroy cell wall structures for direct bacteria killing or induce anti-bacterial metabolites for intracellular bacteria killing have been well understood. However, some critical activity of Se NPs, such as drug delivery, chemosensitization or immunomodulation remain to be further investigated for anti-bacteria treatment.

### Intracellular Mycobacterium Tuberculosis Clearance by Selenium Nanoparticles

Tuberculosis (TB), caused *Mycobacterium tuberculosis* (Mtb), has become one of the top killers among infectious diseases. In recent decades, the occurrence of drug-resistant TB cases become an emerging issue, which requires the development of new alternative treatments beyond the current antibiotics or novel techniques to enhance the efficiency of current antibiotics. Estevez et al. found that Se NPs were able to inhibit the growth of Mtb by damaging their cell envelope integrity, which indicated a new opportunity for the use of Se NPs as antimycobacterial agents by themselves, or for the development of novel nanosystems that combine the action of these Se nanoparticles with other drugs (Estevez et al., 2020).

Up to now, the immune escape of Mtb from phagolysosomal destruction and limited drug delivery into infected cells remain biggest challenges for TB and drug-resistant TB treatments. To synchronously solve the above issues, we combined our decade-long nanotechnology and TB immunology expertise to innovate the macrophage-targeted Se NPs for synergistic antimicrobial and bactericidal destruction of Mtb in host cells (Pi et al., 2020). The mannosylated Se NPs could not only kill Mtb directly, but could also serve as excellent carriers delivering isoniazide specifically into macrophages for enhanced intracellular Mtb killing. More importantly, Se NPs were proved to inhibit Mtb-lysosome escape, dramatically promote the fusion of Mtb into lysosomes to initiate lysosomal clearance of intracellular Mtb. Additionally, more host cell antimicrobial immunity against Mtb, including autophagy, apoptosis and M1 anti-bacterial polarization, were activated for enhanced intracellular Mtb killing. This work demonstrates the potential of Se NPs to establish macrophage-targeted synergetic bactericidal strategy with wide-range innate immunity functions and considerable low cytotoxicity. And also suggest that Se NPs may potentially serve as more effective therapeutics against TB and multidrug-resistant TB.

During the evolution together with human beings in thousands of years, Mtb has become one of the most outstanding and clever bacterial pathogens by its multiple ways to escape from immunological clearance. How to countercharge the immune escape of Mtb remains a substantial challenge for TB or drug-resistant TB treatment. Our works firstly reported that Se NPs, a kind of novel anti-bacteria agent, possessed the ability to regulate host cell immunity for intercepting Mtb immune escape (Pi et al., 2020). These results strongly suggest that Se NPs could not only serve as direct bacteria killing agent or drug delivery system, but could also be used to regulate host immunity for enhanced intracellular bacteria clearance.

### Anti-Viral Infections by Selenium Nanoparticles

Virus, the most dangerous pathogens that causing millions of deaths every year, are now inducing more and more deaths due to the epidemic of COVID-19 virus. How to develop more effective treatments for virus infection becomes the most urgent demand for human health. Selenium has long been found to be directly involved in fighting against viruse infections, such as influenza virus (Yu et al., 2011), Hepatitis virus (Himoto et al., 2011), coxsackie virus (Beck et al., 1995), West Nile virus (Verma et al., 2008), and human immunodeficiency virus (HIV) (Stone et al., 2010). These anti-viral effects are not only associated with the direct virus killing, but also related to its roles in regulating the function of selenoproteins, which introduces Se NPs as ideal antiviral candidates with wide-spectrum antiviral activity (He et al., 2021).

### Selenium Nanoparticles Induced Influenza Virus Inhibition

Different kinds of influenza virus are responsible for the seasonal flu epidemics each year, therefore severely threatening human health. Due to the low toxicity and excellent activity, the antiviral capabilities of Se NPs have attracted increasing attention in recent years. Li et al. introduced oseltamivir decorated Se NPs for H1N1 virus treatment, which significantly interfered the binding of H1N1 influenza virus to host cells through inhibiting the activity of hemagglutinin and neuraminidase (Li et al., 2017). Se NPs could prevent H1N1 from infecting MDCK cells and causing cell apoptosis by blocking chromatin condensation and DNA fragmentation, along with the inhibition of ROS generation and activation of p53 phosphorylation and Akt (Li et al., 2018a). Additionally, they further demonstrated that Se NPs could protect cells and lung tissues from H1N1 virus induced damages by restraining apoptotic signal events (Lin et al., 2018). Moreover, Wang et al. also demonstrated that Se NPs could inhibit H1N1 influenza virus-induced apoptosis by inhibiting ROS-mediated AKT and p53 signaling pathways (Wang C. et al., 2020).

These works collectively suggest that Se NPs could inhibit H1N1 influenza virus-induced apoptosis as novel anti-influenza agents, which might contribute to the control of H1N1 influenza both *in vitro* and *in vivo*. However, it’s worth to note that Amir et al. investigated the efficacy of hexanic extracts of Ficus carica and olive fruit and Se NPs on the immunogenicity of the inactivated avian influenza virus subtype H9N2 in broiler chickens, which indicated that the prepared Se NPs emulsions could elicit a little degree of immunity, but they could not inhibit the anamnestic response and infection (Asl Najjari et al., 2015). In spite of no significant inhibition on H9N2 infection by Se NPs was confirmed by this work, the positive effect on the immunogenicity were found by Se NPs treatment, which as worth for further investigation.

### Enterovirus Inhibition by Selenium Nanoparticles

As the most common pathogens leading to severe cases of hand, foot, and mouth disease (HFMD), Enterovirus 71 (EV71) can induce different clinical symptoms and even death among infants and children under 6 years old. Unfortunately, at present, no effective treatment for EV71 is available, which requires the development of effective treatment strategies. Zhong et al. developed Se NPs as the carrier of oseltamivir to assess the anti-EV71 activity, which apparently enhanced the antiviral effect of oseltamivir to suppress EV71 proliferation and impede cell apoptosis by reducing the caspase-3 activity and ROS generation (Zhong et al., 2019). Lin et al. developed a Se NPs system with siRNA targeting EV71 VP1 gene, which indicated a remarkable interference efficiency in the nerve cell line SK-N-SH and prevented the cells to be infected and restrained host cell apoptosis induced by EV71 (Lin et al., 2020). Taken together, these works demonstrated that Se NPs could serve as a promising drug candidate and drug delivery system against EV71 virus infection, providing the possibilities for the control of EV71 infection.

### Hepatitis Virus Suppression by Selenium Nanoparticles

Hepatitis virus (HV) infection is one of the most serious and prevalent health problems worldwide. The emergence of current anti-HV medications contributes to the slow down of HV induced epidemic, however, some drawbacks including adverse effects and drug resistance are requiring novel agents for more effective and safe treatments. Sodium selenite was found to suppress Hepatitis B virus (HBV) protein expression, transcription, and genome replication in hepatoma cell models in a dose- and time-dependent manner (Cheng et al., 2016), which proved that there is a close relationship between selenium and virus susceptibility. This work firstly confirmed the suppression effects of Selenium on HBV replication and indicated the potentials of Se NPs for HBV treatment.

The prevalence of HBV infection has been significantly reduced by the approved HBV vaccine, which induces strong Th2 responses against that could protect HBV infection. However, there is a vital need to stimulate Th1 prophylactic immune response for more effective control of HBV infection. Mehdi et al. introduced a novel strategy by administration of Se NPs and the HBs antigen vaccine, which could affect lymphocyte proliferation and total antibody responses, and more importantly, could increase IFN-γ level and induce Th1 response (Mahdavi et al., 2017). These immunologic results clearly showed that Se NPs possessed the ability to polarize immune system toward a Th1 pattern and thereby increase the efficacy of vaccines against viral pathogens specifically controlled by cellular immune responses.

### Potential Application of Selenium Nanoparticles in COVID-19 Epidemic

COVID-19 is a widespread, highly contagious and extremely dangerous disease that has caused millions of deaths in the past year. The point-of-care tests for COVID-19 detection is of vital importance for its epidemic prevention and epidemiological investigation. Wang et al. presented a lateral flow immunoassay kit based on Se NPs-modified SARS-CoV-2 nucleoprotein, which detected anti-SARS-CoV-2 IgM and anti-SARS-CoV-2 IgG in human serum by the naked eye within 10 min (Wang Z. et al., 2020). This work demonstrated that the Se NPs based lateral flow kit could conveniently, rapidly, and sensitively detect anti-SARS-CoV-2 IgM and IgG in human serum and blood, highlighting the use of Se NPs for COVID-19 diagnosis and epidemiological investigation.

It’s very interesting that the cure rates in some places are found to be significantly correlated with Selenium intake levels (Moghaddam et al., 2020), which suggests the potential of Selenium for COVID-19 treatment. More importantly, organic Selenium species have been proved to inhibit COVID-19 by covalently binding to the COVID-19 virion Mpro through cell membranes, which results in effective inhibition of COVID-19 infected Vero cells (Jin et al., 2020). Additionally, the ability of Se NPs to inhibit different virus and boost innate and acquired immunity provide more possibilities to use Se NPs as novel anti-viral agents for COVID-19 treatment. However, there are still few works concerning the anti-viral effects of Se NPs against COVID-19, more studies are needed to confirm the role of Se NPs in COVID-19 treatments (He et al., 2021).

### Anti-Fungi Activity of Selenium Nanoparticles

Fungi are ubiquitous and form their own kingdom to threat human health as infections would affect various parts of different species and bodies. Causing a spectrum of diverse diseases, *Candida albicans* is a major opportunistic fungus that very difficult to be controlled. Its biofilms coated by an exopolymeric substance or extracellular polymeric substance matrix can protect the pathogen from adverse environmental conditions, fungicides and hosts’ immunity (Mathé and Van Dijck, 2013). Guisbiers et al. investigated the effects and potential mechanism of Se NPs for inhibiting *Candida albicans* biofilms (Guisbiers et al., 2017). After adhesion on the biofilm, Se NPs could penetrate into the pathogen and then disrupt the cell structure by substituting with sulfur, which induced 50% suppression of *Candida albicans* biofilm at low Se NPs dosage. Aiming at the treatment of Candida and Aspergillus infections, Shakibaie et al. produced biogenic Se NPs to show the strong inhibitory effect on *Candida albicans* and *Aspergillus fumigatus* (Shakibaie et al., 2015). Additionally, Joshi et al. reported that mycogenic Se NPs displayed antifungal activity against *Colletotrichum capsici* and *Alternaria solani*, which highlighted the practical application of Se NPs to manage plant diseases in an ecofriendly manner (Joshi et al., 2019). The broad spectrum antifungal activity of Se NPs against different fungi provides new weapons for the arsenal against fungi induced infectious diseases.

### Anti-Parasite Effects of Selenium Nanoparticles

In addition to possessing antimicrobial effect towards bacteria, virus and fungi, Se NPs also present antiparasitic properties. Biogenic Se NPs were found to display powerful cytotoxicity in killing promastigote and amastigote forms of Leishmania. Major, which suggested that Se NPs could be emerged as a promising therapeutic agent for curing cutaneous leishmaniasis (Beheshti et al., 2013). Se NPs have also been proved to show potent scolicidal effects against Echinococcus granulosus, therefore may be used in Cystic echinococcosis surgery (Mahmoudvand et al., 2014). However, the *in vivo* efficacy of Se NPs remains to be further explored.

Se NPs are found to be more effective than sodium selenite with regard to their anti-coccidial, anti-oxidant, anti-apoptotic and anti-inflammatory role against Eimeria parasite in the jejunum of mice (Alkhudhayri et al., 2018; Alkhudhayri et al., 2020). Furthermore, Dkhil et al. demonstrated the protective roles of Se NPs in mice infected with *Schistosoma mansoni*, indicating that Se NPs could possess therapeutic anti-schistosomal activity in the treatment of intestinal schistosomiasis (Dkhil et al., 2019). Additionally, biogenic Se NPs were also found to show anti-Toxoplasma effects against Toxoplasma gondii in mice with no considerable toxicity, demonstrating the therapeutic effects of Se NPs for toxoplasmosis *in vivo* (Keyhani et al., 2020a; Keyhani et al., 2020b; Shakibaie et al., 2020). These *in vivo* findings along with the previously mentioned *in vitro* results collectively demonstrated that Se NPs could be served as nutritional supplements with powerful anti-parasite effects.

## Conclusion, Perspective and Outlooks

Selenium toxicity can occur with acute or chronic ingestion of excess selenium in humans, and similarly, the excess selenium contents would result in inevitable toxicity against bacteria, virus, fungi and parasite. The strong toxicity of selenium thus makes Se NPs a kind of anti-infection agents with direct killing or inhibition effects on different pathogens. However, it would be very tricky to control the dosages of Se NPs for anti-infection therapy if only the direct killing effects of Se NPs are applied, as the excessive selenium contents might also induce systemic toxicity of normal cells or tissues. Thus, other functions of Se NPs must be involved for more effective anti-infection treatment with low cytotoxicity.

In infectious disease, there are many ways that the host metabolism and immune status can be affected, leading to a dysregulation of redox homeostasis and immunosuppression. Selenium has long been found to be closely associated with different pathogen infections by regulating the antioxidant defense system through selenoprotein functions. As part of antioxidant defense, selenoproteins, such as GPXs and TXNRDs, play an important role in controlling oxidative stress, which therefore results that reduced selenoprotein expression would induce weaken of the defense against infectious diseases (Guillin et al., 2019). Dietary supplementation to provide adequate or supranutritional selenium supply has been proposed to confer health benefits for patients in some important infectious diseases, which indicates the possibility to apply Se NPs as a kind of nutritional supplements for infectious disease defense.

The effective drug delivery and controlled drug release ability of nanosystem are providing more effective tools to enhance the targeting effects of drugs against the specific disease sites. Our previous works have proved that drug delivery system could significantly enhance the intracellular pathogen clearance efficacy of antibiotic by targeting host cells (Pi et al., 2019). Furthermore, we also introduced Se NPs as antibiotic delivery system to promote the intracellular pathogen clearance, which strongly indicated that Se NPs could be also be used as effective drug delivery system to inhibit or kill pathogens in host cells (Pi et al., 2020). These *in vitro* works have confirmed the drug delivery capacity of Se NPs for infectious diseases treatment, however, more *in vivo* works are needed for their future applications.

Up to now, most therapeutics against infectious diseases are focusing on the antibiotic treatments, however, the increasing emergence of drug-resistant mutants or multiple drug-resistant mutants requires novel treatments rather than more antibiotics that might worsen the drug-resistance conditions. Immune therapy is now serving as one of the most effective treatments in some important disease, such as tumor. It’s very attractive that Selenium status may affect the function of cells both in adaptive and innate immunity. We have recently demonstrated the ability of Se NPs to regulate host immunity against intracellular pathogens, which dramatically countercharge the immune escape of intracellular pathogens, such as lysosomal escape, host cell apoptosis/autophagy and macrophage polarization (Pi et al., 2020). This means that Se NPs could be served as effective immunomodulation agents for innate immunity regulations against infectious diseases.

Supranutritional selenium intake was shown to regulate adaptive immunity by favoring proliferation and differentiation of activated CD4-positive T cells toward Th1 cells, which play important roles in infectious disease defense (Steinbrenner et al., 2015). Although there are few works to elaborate the effects of Se NPs against infectious diseases by regulating adaptive immunity, lots works have also demonstrated the potentials of Se NPs to manipulate adaptive immunity against other diseases (Hu et al., 2019). It would be an attractive topic to explore the effects of Se NPs on adaptive immunity, which would further extend the application of Se NPs for more effective infectious disease treatments.

Taking the advantages of direct inhibition/killing effects on pathogens, targeted drug delivery against host cells or disease sites, chemosensitization effects on the current drugs, and the innate and adaptive immunity regulation effects, Se NPs are expected to be served as effective anti-infectious agents simultaneously manipulating the above activities. Considering the low toxicity, antioxidant and immunity regulation capabilities and other merits of Se NPs, it is desirable and reasonable to further explore the effects and mechanisms of Se NPs against different pathogens. With the development of nanotechniques, we hope that Se NPs can play more and more important roles in fighting against infectious pathogens, which would finally benefit the diagnosis, prevention and treatment of infectious diseases.

## Future Directions

As a kind of novel nanomaterials, Se NPs have drawn increasing attentions to address the dilemma of antibiotic resistance, thus showing attractive potentials for future clinical infectious diseases treatment. However, there are still some inevitable challenges that need to be addressed before their clinical transformations. During these challenges for Se NPs, the most urgent issue is the biocompability, which is the ability of a material being compatible with living tissue. Ideal biocompatible nanomaterials would not produce unexpected toxicity or immunological response when exposed to the body or bodily fluids. However, the toxicity of excess selenium is a dangerous assumption that Se NPs may introduce. The toxicity of Se NPs have been widely reported for anticancer or anti-infection treatment, however, the toxicity of Se NPs against normal cells or tissues remains to be further investigated. Thus, it would be important to understand the bridge between the Selenium and Se NPs, especially for their molecular events that are responsible for the therapeutic differences and toxicity effects that are critical for their biocompability. Otherwise, the degradation of Se NPs in body is still unclear, which might introduce unknown toxicity after long-term administration. Thus, more concerns about the degradation of Se NPs after long-term administration should be paid to verify the safety of Se NPs for clinical uses. Therefore, how to develop functional Se NPs with good biocompability and degradation property would be the most critical issues for the further clinical application of Se NPs against different infectious diseases.

Another critical issue for the further studies of Se NPs would be focused on the exploration of their anti-infectious mechanisms, especially their effects and mechanism on immune regulations. The direct killing effects of Se NPs against different pathogens are the mostly investigated parts for their potential anti-infection applications. However, as widely known, one of the most important issues in infectious diseases would be the immune responses for infection controls. The immunity regulation functions of Selenium are thought to be closely associated with selenoproteins, which play critical roles in both metabolism and immune system. However, how selenium from Se NPs affect the immune responses by regulating selenoprotein activity remains to be further investigated. And in our opinion, Se NPs may also strongly influence the phagocyte functions to further regulate the immune responses. Thus, it would be important to explore the effects of Se NPs on phagocyte functions when they are used for infectious disease treatment, which may also activate anti-infection immunity for infections control.
